# Prognostic analysis of cutaneous Kaposi sarcoma based on a competing risk model

**DOI:** 10.1038/s41598-023-44800-5

**Published:** 2023-10-16

**Authors:** Bei Qian, Ying Qian, Peng Xiao, Liang Guo

**Affiliations:** 1 Department of Thyroid and Breast Surgery, Union Hospital, Tongji Medical College, Huazhong University of Science and Technology, Wuhan, 430022 Hubei China; 2 Department of Pharmacy, Jingzhou Hospital, Yangtze University, Jingzhou, 434020 Hubei China; 3 Department of Plastic Surgery, Union Hospital, Tongji Medical College, Huazhong University of Science and Technology, Wuhan, 430022 Hubei China

**Keywords:** Cancer epidemiology, Cancer prevention, Cancer screening, Skin cancer

## Abstract

The data regarding the prognosis of cutaneous Kaposi sarcoma (KS) was limited. The current study aimed to explore the risk factors and develop a predictive model for the prognosis of cutaneous KS patients. Data were extracted from Surveillance, Epidemiology, and End Results database from 2000 to 2018 and randomly divided into training and validation cohort. The Kaplan–Meier analysis, cumulative incidence function based on the competing risk model and Fine–Gray multivariable regression model was used to identify the prognostic factors and then construct a 5-, 10-, and 15-year KS-specific death (KSSD) nomogram for patients. The concordance index (C-index), area under the curve (AUC) of operating characteristics and calibration plots were used to evaluate the performance of the model. The clinical utility of the model was measured by decision curve analysis (DCA). In 2257 cutaneous KS patients identified from database, the overall median survival time was about 13 years. Radiotherapy (*p* = 0.013) and surgery (*p* < 0.001) could lower the KSSD, while chemotherapy (*p* = 0.042) and surgery (*p* < 0.001) could increase the overall survival (OS) of patients with metastatic and localized lesions, respectively. Race, number of lesions, surgery, extent of disease, year of diagnosis and age were identified as risk factors associated with cutaneous KS-specific survival. Performance of the nomogram was validated by calibration and discrimination, with C‐index values of 0.709 and AUC for 5-, 10-, and 15-year-KSSD of 0.739, 0.728 and 0.725 respectively. DCA indicated that the nomogram had good net benefits in clinical scenarios. Using a competing-risk model, this study firstly identified the prognostic factors, and constructed a validated nomogram to provide individualized assessment and reliable prognostic prediction for cutaneous KS patients.

## Introduction

Kaposi Sarcoma (KS) is a rare angioproliferative neoplasm of endothelial origin caused by the infection with human herpes virus 8 (HHV-8), also known as Kaposi sarcoma-associated herpes virus (KSHV)^1^, which is spread by saliva and sexual contact^2^. Cutaneous KS most frequently manifests as scattered pink to purple patches and papules, or rapidly progressive, multicentric, ulcerated plaques and nodules ranging from several millimeters to centimeters^3,4^. According to different groups of affected people, KS is divided into four main epidemiological subtypes: classic or Mediterranean (occurs in middle-aged and elderly people without HIV infection), endemic or African (affects people without HIV infection in sub-Saharan Africa), iatrogenic or post-transplantation (occurs in people after solid organ transplantation), and epidemic or AIDS-related (people with HIV infection)^5,6^. KS is considered to be the second most common tumor among people living with HIV (PLWH) in the United States^7^. A definitive diagnosis relies on a biopsy of skin nodules. The histological features include inflammatory infiltration, aberrant vascular proliferation, and hyperproliferation of spindle cells^8^. The presence of HHV8 antigen observed by immunohistochemical detection may aid the diagnosis^9^. The goal of KS therapy is to induce sustained remission. Local treatment such as surgery, cryotherapy and brachytherapy is recommended for localized diseases, while systemic treatment is indicated in extensive or painful cutaneous diseases, visceral diseases, or cases of rapid progression^10^. In patients with AIDS-related KS, antiretroviral therapy (ART) has been proven to be the cornerstone of treatment to bolster the immune system, with a 20–80% response rate^11^. For iatrogenic KS, the initial treatment aimed to alter the immunosuppressive state. For other subtypes of KS or advanced KS, although chemotherapy may be not curable, it is necessary, such as liposomal doxorubicin and paclitaxel^12,13^. Meanwhile, the efficacy of new therapies such as immune checkpoint inhibitor PD-1/L1 in KS has been reported^14,15^. However, due to the lack of effective large cohort studies or prospective researches, it is still difficult to counsel patients with cutaneous KS on their treatment strategy and prognosis.

Currently, nomogram, a simplified numerical model for statistical predictions, have been considered reliable tools to predict the prognosis of cancer patients^16,17^. Thus, in the present study, we aimed to explore the prognostic factors of cutaneous KS using a large dataset from the Surveillance, Epidemiology and End Results (SEER) database. Meanwhile, a nomogram based on the competing risk model would be constructed to predict the long-term disease specific survival outcomes of patients with cutaneous KS.

## Patients and methods

### Data source and patient selection

Data on patients with cutaneous KS were obtained from the SEER database (https://seer.cancer.gov/) consisting of 18 population-based cancer registries, corresponding to the period 2000–2018. The extraction conditions were as follows: “primary sites: C44.9-Skin, NOS”; “Behavior code: Malignant”; “Histologic type ICD-O-3: 9140/3” and “diagnosis year: 2000 to 2018”. The extracted variables included: patient ID, sex, age at diagnosis, year of diagnosis, race record, ICD-O-3 His/behave, Laterality, Combined Summary Stage (extent of disease), Surgery of primary site, Radiotherapy recode, Chemotherapy recode, distant metastases record, number of lesions, survival months, SEER cause-specific death classification, vital status recode (study cut-off used), SEER registry and marital status at diagnosis. The exclusion criteria were as follows: (1) nonpathological diagnosis; (2) survival time unknown and (3) cause of death unknown. Clinicopathological and demographic data were collected for all eligible cases. This study was exempt from the approval processes of the Institutional Review Boards because the SEER database patient information was de-identified.

### Cohort definition and variable recodes

A total of 2257 eligible cutaneous KS patients were randomly divided into training cohort (n = 1808) and validation cohort (n = 449) using the R function “createDataPartition” at a ratio of 8:2 to establish and validate the nomogram respectively. The variables analyzed included: all factors, gender (female or male), age (≥ 60 or < 60), year of diagnosis (≥ 2008 or < 2008), race (black, white or other), Spanish-Hispanic-Latino (yes or no), extent of disease (localized, distant, regional or other), multifocality (yes or no), surgery (yes or no), radiotherapy (yes or no), chemotherapy (yes or no) and marriage (yes or no). The cut‐off point of Continuous variable such as age and year of diagnosis for risk stratifications was generated by the “surv_cutpoint” function of the “Survminer” R package.

Overall Survival (OS) of patients in the different risk groups were analyzed using the Kaplan–Meier method, and the survival differences were compared using a log-rank test. Additionally, further subgroup analysis of the impact of gender, age group, multifocal, surgery, radiotherapy and chemotherapy on the OS of the cutaneous KS patients was performed according to the extent of disease.

### Construction of the nomogram

The main endpoint in the present study was KS-specific death (KSSD). KSSD and other causes of death (Non-KSSD) were two events in our competing-risk analysis. The competitive risk analysis model was applied to control the risk of other cause of death while comparing the difference in the risk of KSSD. The CIF (cumulative incidence function) was used to describe the probability of death and Gray’s test to applied to compare CIF across categories^18^. We calculated the CIF for each event and plotted CIF curves. simple competing risk model was used to check each factor’s power in predicting KSSD. Variables considered clinically relevant or significant differences in simple analysis (*p* < 0.2) would be introduced into the multiple competitive risk analysis model. variables with *p* values < 0.05 in the multivariable analysis were included in the final model. Based on the coefficients from the competing risks regression models, a nomogram based on Fine and Gray’s model was built by the R packages “mstate” and “regplot”^19^. Meanwhile, a nomogram based on the multiple COX proportional hazard model was constructed to compare the two results.

### Discrimination and calibration of the nomogram

The concordance (C)-index was applied to evaluate discrimination^20^ and calibration was assessed using a calibration plot. 1000 bootstraps were used for plotting the calibration curve and calculating the C-index. The C index ranges from 0.5 to 1, where 1 means perfect discrimination and 0.5 means no discrimination. The calibration plot showed the correlation between the predicted probability and the frequency of the observed outcome. The standard curve was a straight line with a slope of 1 passing through the origin of the coordinate axis. The closer the calibration curve was to the standard curve, the better the predictive ability of the nomogram^21^. Decision curve analysis (DCA) was used to evaluate the clinical utility and net benefits of competitive risk models^22^. The curves of treat‐all‐patients scheme (representing the highest clinical costs) and the treat‐none scheme (representing no clinical benefit) were plotted as two references^23^.

### Statistical analysis

Continuous variables conforming to the normal distribution were expressed as the mean ± standard deviation (SD), while continuous variables with a skewed distribution were presented as the median and interquartile range (IQR). Categorical variables were shown as frequencies and their proportions. Chi-square test or Fisher’s exact test was used to analyze the statistical difference of the categorical covariates distribution between the training cohort and the validation cohort, and Mann–Whitney test for continuous and non-normally distributed covariates. A two-tailed *p* < 0.05 was considered to be statistically significant. All statistical analyses and visualization were performed by using R studio statistical software version 1.4.1717 (https://www.r-project.org).

## Results

### Demographics and clinicopathological characteristics of patients

**Figure 1:**
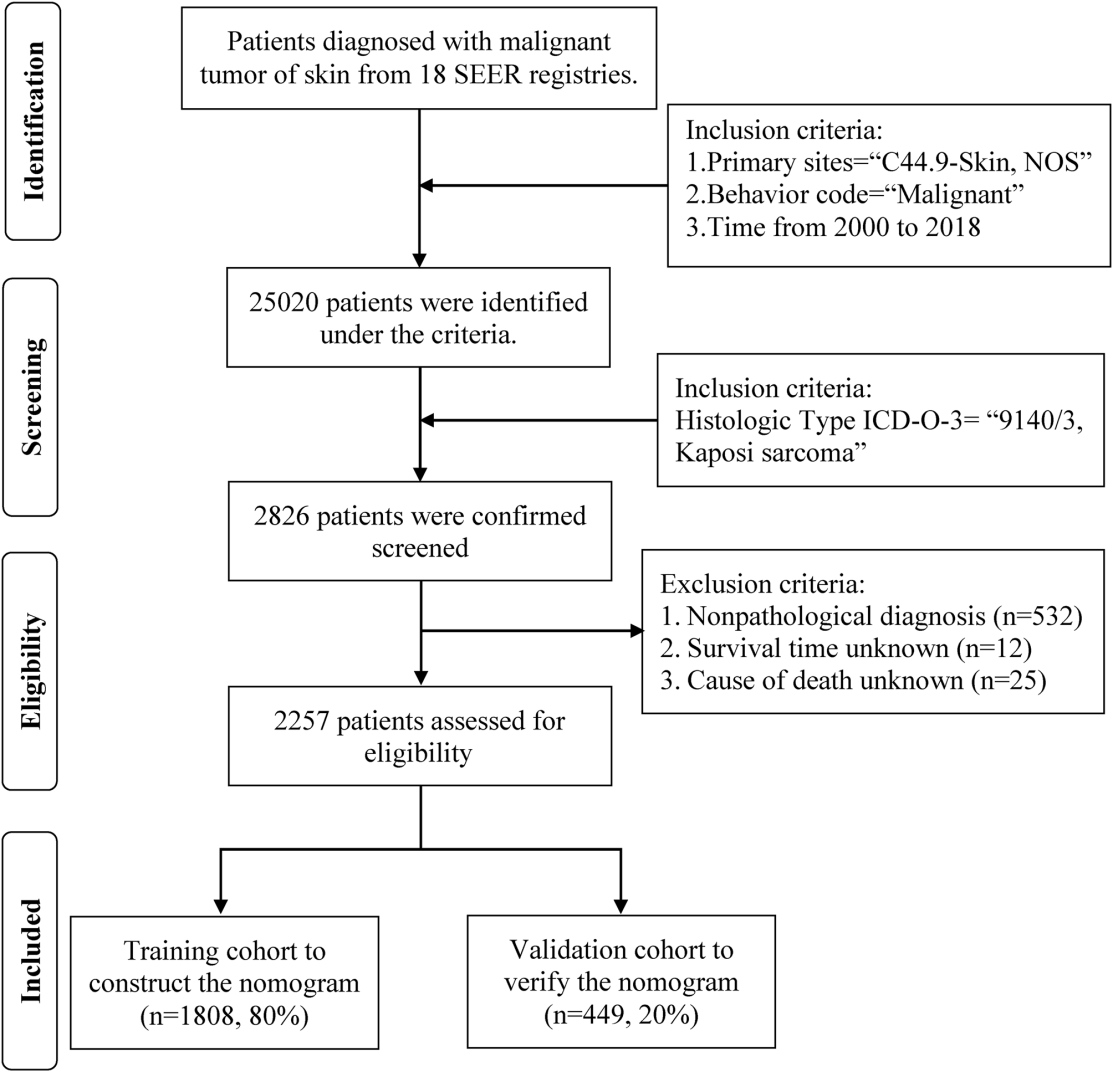
Flow diagram presenting the screening process in the SEER database.

**Figure 2:**
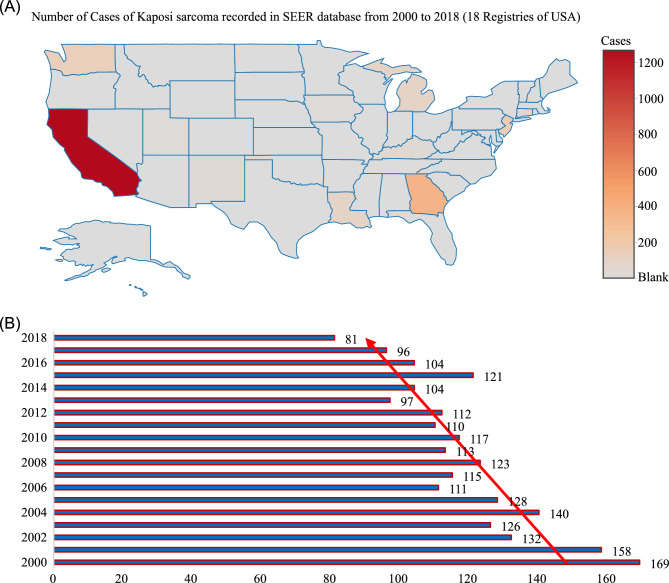
The regional distribution and year of diagnosis characteristics of all cutaneous KS cases from SEER database consisting of 18 population-based cancer registries; KS: Kaposi sarcoma.

A total of 2257 patients with cutaneous KS were identified from the SEER database from 2000 to 2018, of which 1808 (80%) were assigned to the training cohort, and 449 (20%) cases were assigned to the validation cohort. Figure 1 presented the detailed screening process in the SEER database. The median age at diagnosis was 40 years (IQR: 33–49), and the median follow-up of the whole study cohort was 47 months (IQR: 8–120). A larger proportion of cutaneous KS patients were aged under 60 years (1960, 86.8%), male (2146, 95.1%), single (1736, 76.9%), Non-Spanish-Hispanic-Latino (1682, 74.5%), and non-multifocality (1866, 82.7%). Among all patients, 283 (12.5%) patients performed surgery, 158 (7.0%) and 815 (36.1%) patients undergone radiotherapy and chemotherapy respectively. The distribution of race was as follows: 622 (27.6%) were black, 1458 (64.6%) were white, 177 (7.8%) were other race. Most patients were diagnosed with a localized disease (887, 39.3%), followed by regional (667, 29.6%), distant (125, 5.5%), and other (578, 25.6%). The distant metastatic organs of cutaneous KS include 19 (0.8%) cases of lung, 7 (0.3%) cases of liver, 11 (0.5%) cases of distant lymph nodes, and 7 (0.3%) cases of bone. The baseline demographics and clinicopathological characteristics of all eligible patients were presented in Table 1. The patient characteristics of the training (n = 1808) and validation cohorts (n = 449) were concluded in Table 2. Since all cases were randomly assigned to the training and the validation cohort, there was no statistical difference between the variables of the two cohorts (all the P values > 0.05). The regional distribution and year of diagnosis characteristics of all acquired cases were visualized in Fig. 2A, B, respectively. California has the most confirmed cases. There seemed to be a downward trend in the annual number of cases over the past two decades, which may reflect a decline in the incidence of cutaneous KS.

**Table 1:** Demographics and clinicopathological characteristics of patients with cutaneous Kaposi sarcoma.

Characteristic	Level	Overall
N		2257
Age (median [IQR])		40.00 [33.00, 49.00]
Age group (%)	< 60	1960 (86.8)
	≥ 60	297 (13.2)
Gender (%)	Female	111 (4.9)
	Male	2146 (95.1)
Diagnosis of year (%)	< 2008	1079 (47.8)
	≥ 2008	1178 (52.2)
Race (%)	Black	622 (27.6)
	Other	177 (7.8)
	White	1458 (64.6)
Spanish-Hispanic-Latino (%)	No	1682 (74.5)
	Yes	575 (25.5)
Extent of disease (%)	Distant	125 (5.5)
	Localized	887 (39.3)
	Other	578 (25.6)
	Regional	667 (29.6)
Surgery (%)	No	1963 (87.0)
	Other	11 (0.5)
	Yes	283 (12.5)
Radiotherapy (%)	No	2099 (93.0)
	Yes	158 (7.0)
Chemotherapy (%)	No	1442 (63.9)
	Yes	815 (36.1)
KSSD (%)	No	1534 (68.0)
	Yes	723 (32.0)
Non-KSSD (%)	No	1999 (88.6)
	Yes	258 (11.4)
Follow up months (median [IQR])		47.00 [8.00, 120.00]
Number of lesions (median [IQR])		1.00 [1.00, 1.00]
Multifocality (%)	No	1866 (82.7)
	Yes	391 (17.3)
Marriage (%)	Married	266 (11.8)
	Single	1736 (76.9)
	Unknown	255 (11.3)
Distant metastases (%)	Lung	19 (0.8)
	Bone	7 (0.3%)
	Liver	7 (0.3%)
	Distant lymph node	11 (0.5%)
	Other organ	15 (0.7%)

*KSSD* Kaposi sarcoma-specific death, *IQR* interquartile range, *NOS* not otherwise specified, *mm* millimetre.

**Table 2:** Baseline clinicopathological characteristics and treatment experience of patients in the training and validation cohort.

Characteristic	Level	Training cohort	Validation cohort	*p* value
N		1808	449	
Age (median [IQR])		40.0 [33.0, 49.0]	41.0 [34.0, 49.0]	0.57
Age group (%)	< 60	1568 (86.7)	392 (87.3)	0.75
	≥ 60	240 (13.3)	57 (12.7)	
Gender (%)	Female	97 (5.4)	14 (3.1)	0.05
	Male	1711 (94.6)	435 (96.9)	
Diagnosis of year (%)	< 2008	875 (48.4)	204 (45.4)	0.26
	≥ 2008	933 (51.6)	245 (54.6)	
Race (%)	Black	514 (28.4)	108 (24.1)	0.09
	Other	134 (7.4)	43 ( 9.6)	
	White	1160 (64.2)	298 (66.4)	
Spanish (%)	No	1348 (74.6)	334 (74.4)	0.94
	Yes	460 (25.4)	115 (25.6)	
Stage (%)	Distant	111 ( 6.1)	14 ( 3.1)	0.05
	Localized	694 (38.4)	193 (43.0)	
	Other	464 (25.7)	114 (25.4)	
	Regional	539 (29.8)	128 (28.5)	
Surgery (%)	No	1582 (87.5)	381 (84.9)	0.17
	Other	10 ( 0.6)	1 ( 0.2)	
	Yes	216 (11.9)	67 (14.9)	
Radiation (%)	No	1688 (93.4)	411 (91.5)	0.18
	Yes	120 ( 6.6)	38 ( 8.5)	
Chemotherapy (%)	No	1150 (63.6)	292 (65.0)	0.57
	Yes	658 (36.4)	157 (35.0)	
KSSD (%)	No	1224 (67.7)	310 (69.0)	0.59
	Yes	584 (32.3)	139 (31.0)	
Non-KSSD (%)	No	1596 (88.3)	403 (89.8)	0.38
	Yes	212 (11.7)	46 (10.2)	
Number of lesions (median [IQR])		1.0 [1.0, 1.0]	1.0 [1.0, 1.0]	0.15
Multifocality (%)	No	1485 (82.1)	381 (84.9)	0.17
	Yes	323 (17.9)	68 (15.1)	
Marriage (%)	Married	225 (12.4)	41 ( 9.1)	0.13
	Single	1377 (76.2)	359 (80.0)	
	Unknown	206 (11.4)	49 (10.9)	

*KSSD* Kaposi sarcoma-specific death, *IQR* interquartile range, *NOS* not otherwise specified, *mm* millimeter.

### Kaplan–Meier analysis of OS

**Figure 3:**
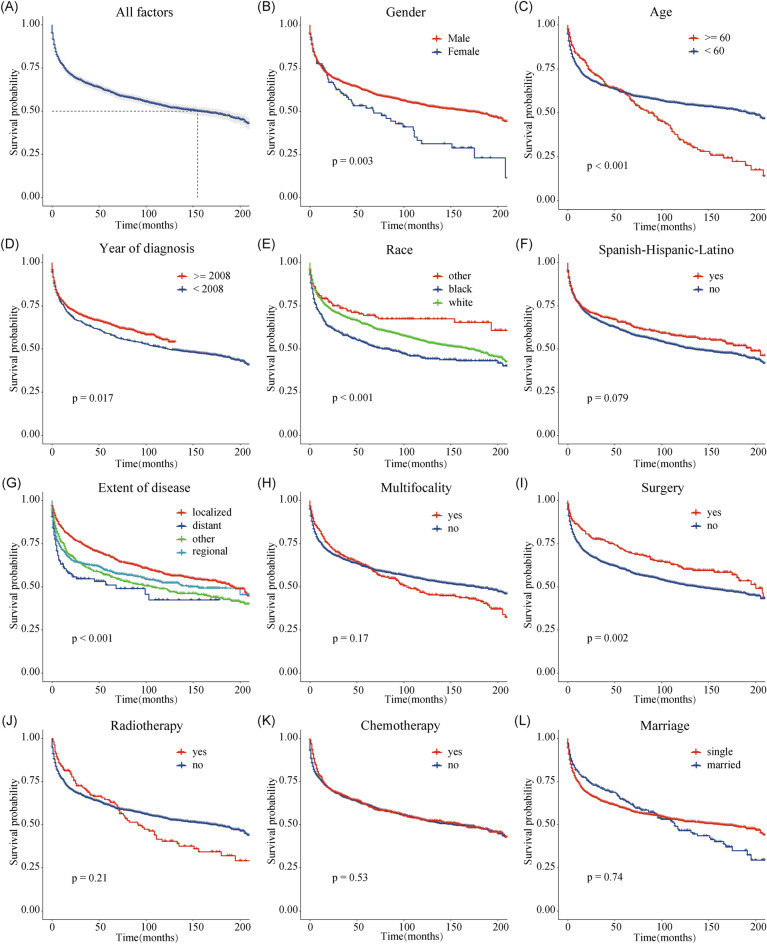
Kaplan–Meier curves of OS for cutaneous KS patients at different stages or with different characteristics. (**A**) All factors; (**B**) Gender; (**C**) Age; (**D**) Year of diagnosis; (**E**) Race; (**F**) Spanish-Hispanic-Latino; (**G**) Extent of disease; (**H**) Multifocality; (**I**) Surgery; (**J**) Radiotherapy; (**K**) Chemotherapy; (**L**) Marriage. *OS* overall survival, *KS* Kaposi sarcoma.

**Figure 4:**
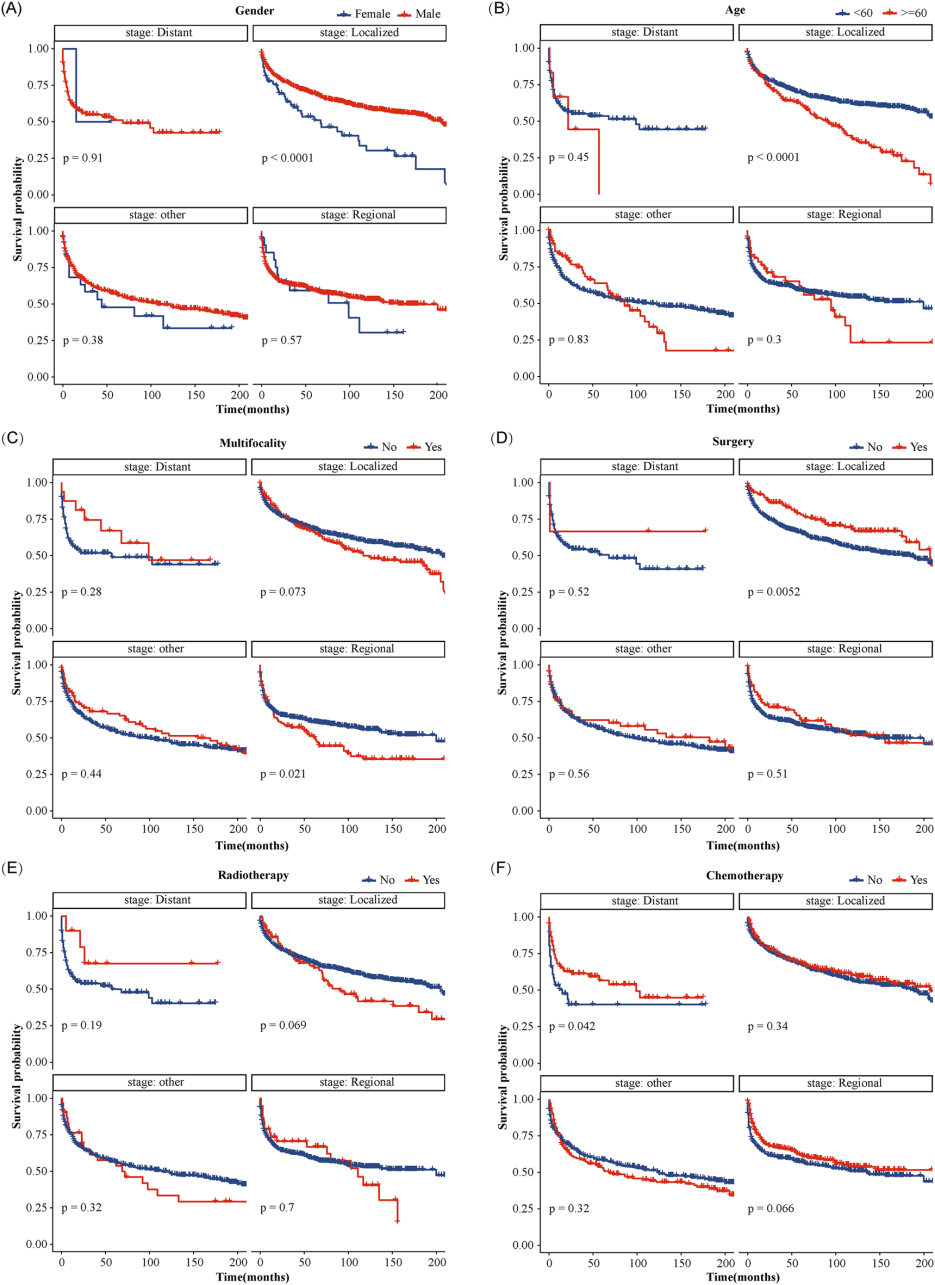
Kaplan–Meier subgroup analysis of OS for cutaneous KS patients with different characteristics. (**A**) Gender; (**B**) Age; (**C**) Multifocality; (**D**) Surgery; (**E**) Radiotherapy; (**F**) Chemotherapy. *OS* overall survival, *KS* Kaposi sarcoma.

A total of 981 (43.5%) patients died in this study, and 723 (32.0%) of them had a KSSD, while 258 (11.4%) did not. The results of OS analysis for each variable were presented in Fig. 3, which showed that men (*p* = 0.003), age < 60 (*p* < 0.001), White race (*p* < 0.001), year of diagnosis after 2008 (*p* = 0.017), surgery performed (*p* = 0.002) were associated with better OS for the patients. In terms of extent of disease, patients with a localized lesions had the best prognosis, followed by regional lesions and distant metastatic lesions (*p* < 0.001). Figure 3A indicated that nearly half of cutaneous KS patients died within 13 years. Further subgroup survival analysis (Fig. 4) suggested that for regional or metastatic lesions, age or gender did not show statistical differences (All p value > 0.05). For localized lesions, men (*p* < 0.0001) or patients younger than 60 years old (*p* < 0.0001) have a better OS. For regional lesions, multifocality may be associated with worse OS (*p* = 0.021). The benefits of surgery and chemotherapy were manifested in localized (*p* = 0.0052) and distant metastatic lesions (*p* = 0.042), respectively. For regional lesions, although chemotherapy seemed to be associated with better OS, no statistical significance was shown in this study (*p* = 0.066). No statistical difference was found between radiotherapy and OS (Fig. 4E, all *p* value > 0.05).

### Nomogram variable screening by competing risk analysis

**Figure 5:**
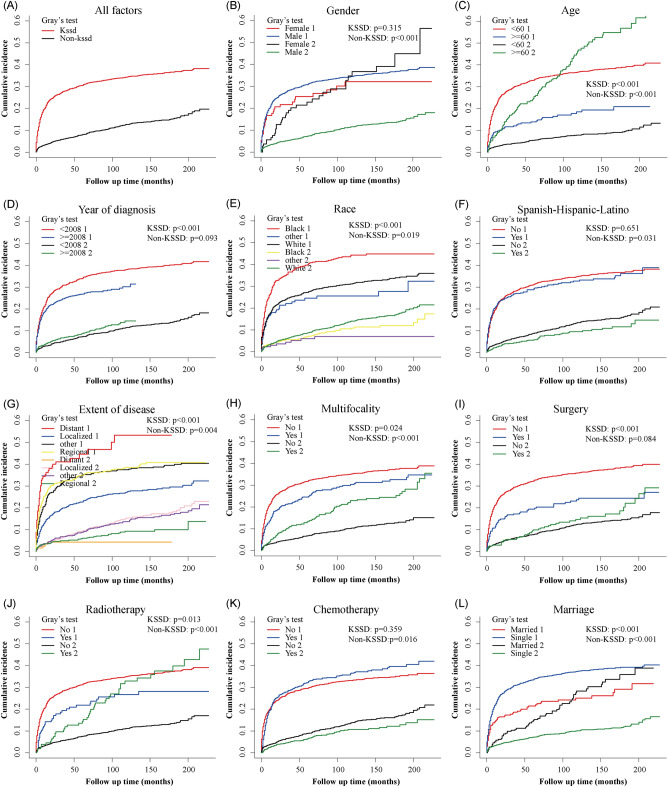
Cumulative KS-specific and competing mortality stratified by patient characteristics (“1” indicated KS-specific death, KSSD; “2” indicated other cause of death, Non-KSSD). (**A**) All factors; (**B**) Gender; (**C**) Age; (**D**) Year of diagnosis; (**E**) Race; (**F**) Spanish-Hispanic-Latino; (**G**) Extent of disease; (**H**) Multifocality; (**I**) Surgery; (**J**) Radiotherapy; (**K**) Chemotherapy; (**L**) Marriage. *KS* Kaposi sarcoma.

The CIF curves of KSSD and Non-KSSD by different clinicopathological characteristics were shown in Fig. 5. Overall, the incidence of KSSD was higher than that of Non-KSSD (Fig. 5A). Patients with a higher CIF of KSSD were those with characteristics of younger age (*p* < 0.001), diagnosed before 2008 (*p* < 0.001), non-multifocality (*p* = 0.024), without surgery (*p* < 0.001), without radiotherapy (*p* = 0.013) and single (*p* < 0.001). Also, different races (*p* < 0.001) and different extent of disease (*p* < 0.001) showed statistical differences in KSSD. However, the CIF of KSSD did not differ significantly in gender and between those with and without chemotherapy. The simple and multiple analysis of the variables by competing risks regression suggested that age (< 60 vs. ≥ 60: HR 2.75, 95% confidence interval [CI] 1.88–4.02, *p* < 0.001), year of diagnosis (HR 0.96, 95%CI 0.95–0.98, *p* < 0.001), race (white vs. black: HR 0.77, 95% CI 0.63–0.93, *p* = 0.008), extent of disease (regional vs. distant: HR 065, 95% CI 0.45–0.96, *p* = 0.028; localized vs. distant: HR 0.39, 95%CI 0.26–0.57, *p* < 0.001; other vs. distant: HR 0.80, 95% CI 0.67–0.96, *p* = 0.016), surgery (yes vs. no: HR 0.71, 95% CI 0.51–0.98, *p* = 0.037), number of lesions (HR 0.80, 95% CI 0.67–0.96, *p* = 0.016) were the independent t prognosis factors of KSSD (Table 3).

**Table 3:** Univariate and Multivariate competing risk model for KSSD in patients with Kaposi sarcoma.

Characteristic	Simple competing risk model	Multiple competing risk model		
	*p* value	Coefficient	HR (95%CI)	*p* value
Age	**0.002**			
≥ 60		Ref.	Ref.	
< 60		1.012	2.75 (1.88–4.02)	< **0.001**
Gender	0.710			
Male		–	–	
Female		–	–	
Year of diagnosis	**0.019**	− 0.036	0.96 (0.95–0.98)	< **0.001**
Race	**0.002**			
Black		Ref.	Ref.	
White		− 0.264	0.77 (0.63–0.93)	**0.008**
Other		− 0.240	0.79 (0.52–1.19)	0.257
Spanish-Hispanic-Latino	0.240	–	–	
No		–	–	
Yes		–	–	
Extent of disease	**0.003**			
Distant		Ref.	Ref.	
Regional		− 0.423	0.65 (0.45–0.96)	**0.028**
Localized		− 0.952	0.39 (0.26–0.57)	< **0.001**
Other		− 0.690	0.50 (0.34–0.74)	**0.001**
Number of lesions	0.130	− 0.218	0.80 (0.67–0.96)	**0.016**
Surgery	**0.003**			
No		Ref.	Ref.	
Yes		− 0.343	0.71 (0.51–0.98)	**0.037**
Radiotherapy	0.088			
No		Ref.	Ref.	
Yes		− 0.285	0.75 (0.50–1.13)	0.168
Chemotherapy	0.820			
No		–	–	
Yes		–	–	
Marriage	0.220			
Married		–	–	
Single		–	–	

*KS* Kaposi sarcoma, *KSSD* Kaposi sarcoma-specific death, *HR* hazard ratio, *CI* confidence interval.

Significant values are in bold.

### Construction of competing risks regression nomogram model

**Figure 6:**
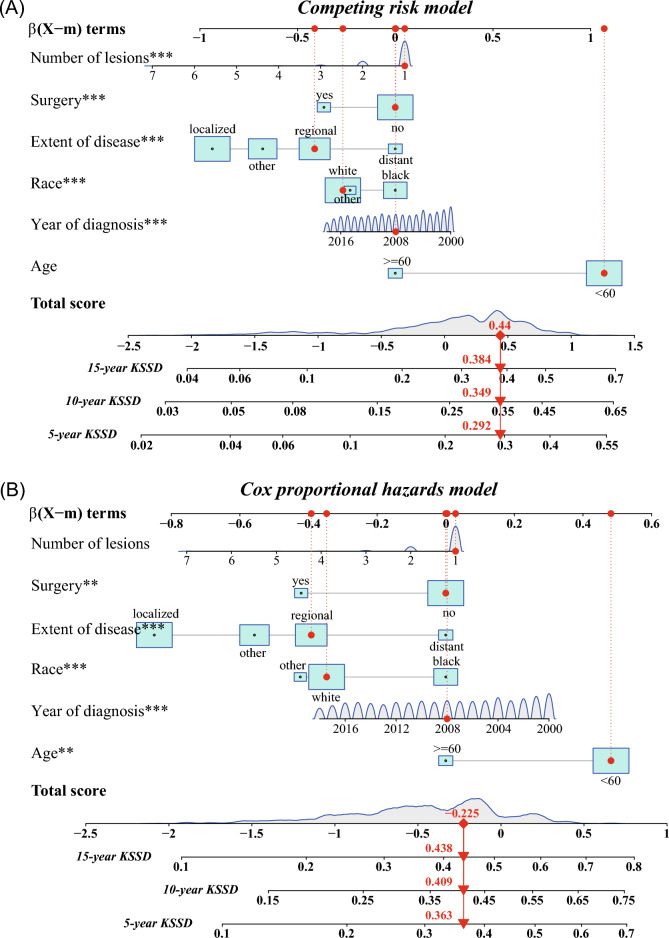
The nomogram for predicting 5-, 10- and 15-year KSSD of cutaneous KS patients. KS-specific mortality was determined by adding up the scores of all variables and drawing a vertical line between the total point scale and the probability of death scale. (**A**) The nomogram based on the competing risk model; (**B**) The nomogram based on the Cox proportional hazard model. *KSSD* Kaposi sarcoma-specific death.

All statistically significant clinical parameters after multiple analysis, and clear clinical prognosis-related factors including age, year of diagnosis, race, extent of disease, number of lesions and surgery were used for the construction of nomogram. Therefore, a nomogram established by competing risks regression models were constructed to calculate the 5-year, 10-year and 15-year cumulative KSSD probabilities in patients with cutaneous KS (Fig. 6A). The C-index of this nomogram for the training cohort was 0.709, demonstrating good accuracy for KSSD prediction. At the same time, another nomogram with a C-index of 0.625 (Fig. 6B) based on the multiple Cox regression model was developed to compare the KSSD difference with the above model. For each patient, first located the values of different variables in the corresponding variable row, and then added the scores of all the variables to get the total score. Drew a vertical line pointing to the bottom scale according to the total score to get the corresponding KSSD possibility. For example, a for a given patient (Patient ID = 35767086), the nomogram based on the competing risks regression models indicated that this cutaneous KS patient may had a 5-year KSSD of 29.2%, a 10-year KSSD of 34.9%, and a 15-year KSSD of 38.4%. However, the nomogram based on the Cox regression model suggested that the corresponding values were 36.3%, 40.9%, and 43.8%, which were all higher than the values based on the competing risk model.

### Validation and calibration of the nomogram

**Figure 7:**
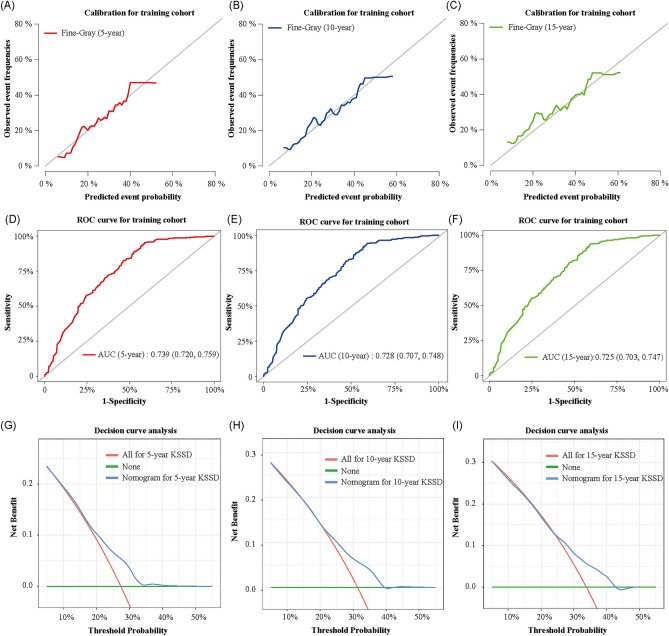
Discrimination and calibration validation of the nomogram model. (**A–C**) The 5-, 10- and 15-year calibration curves for the training cohort. The X-axes indicated the mean predicted probability of KSSD according to the prediction model, and the Y-axes represented the observed cumulative incidence of Kaposi sarcoma-specific death. The gray diagonal line indicated that the predicted value and the observed value were equal. (**D–F**) The area under the curve (AUC) of operating characteristics curve (ROC) for 5-, 10-, and 15-year-KSSD respectively. (**G–I**) Decision curve analysis of the nomogram. Horizontal green lines assumed no cases would experience the KSSD; Red lines assumed all cases would experience the KSSD. Blue lines represented the clinical net benefits across a range of threshold probabilities, within which applying the nomogram to predict the KSSD gained more benefit than the hypothetical treat-all or treat-none scenarios, *KSSD *Kaposi sarcoma-specific death.

The calibration plot assessing the agreement between the values predicted by the nomogram and the observed outcomes in training cohort was presented in Fig. 7A–C and validation cohort in supplementary Fig. S1. The closer the calibration curve was to the 45-degree diagonal, the better the calibration of the established nomogram. And the AUC for 5-, 10-, and 15-year-KSSD was 0.739 (0.72, 0.759), 0.728 (0.707, 0.748) and 0.725 (0.703, 0.747) respectively (Fig. 7D–F). In addition, the DCA curves used to evaluate the clinical utility and net benefits of the nomogram was shown in Fig. 7G–I. When the threshold probabilities of 5-year, 10-year, and 15-year KSSD were in the range of 20–41%, 25–45%, and 27–47% respectively, the net clinical benefit from the competitive risk model was higher than the hypothetical non-screening or all screening scenarios.

## Discussion

Cutaneous KS was a rare cancer, epidemiological research into it was therefore scarce. So far, there was no universally accepted staging classification for the cutaneous KS^24^,which illustrated the necessity to explore the prognostic factors based on a registered population and construct a nomogram for improving the individualized assessment and prediction capabilities for patients. Thus, this study aimed to explore the risk factors of cutaneous KS by screening patients from the latest population-based database between 2000 and 2018. Subsequently, regarding the death as a competing factor, a nomogram based on the competing risk model was developed to predict the 5-,10-, and 15-year KSSD of cutaneous KS patients.

Among the states included in the registration, California had the most recorded cases, followed by Georgia and New Jersey (Fig. 2A), which may due to a higher proportion of MSM (Men who have sex with men) among HIV population in these states^25^. Meanwhile, the annual number of cutaneous KS cases recorded in 18 registered areas in the United States seemed to show a downward trend, which may roughly reflect changes in the overall incidence from 2000 to 2018 (Fig. 2B). It was consistent with the previous reports in the literature^26^. Additionally, other countries also showed a decrease in the incidence of KS such as Italy^27^ and Switzerland^28^, etc. Scholars attributed this decrease to the introduction of antiretroviral therapy (ART), which had led to fewer HIV-infected patients developing KS^29,30^.

Preliminary analysis showed that nearly 50% of cutaneous KS patients died about 13 years after diagnosis (Fig. 3A). However, a study on 204 classic KS from Italian population-based cancer registries showed a median survival of 9.4 years^31^. From the Fig. 3A, the result was similar to the 67% 5-year net survival reported in the literature, which lower than 79% in Europe^32^. Male (vs. female) and patients with a diagnosis age less than 60 (vs. > 60 years) had better OS (Fig. 3B, C), and further subgroup analysis suggested that this difference was mainly manifested in cases with localized lesions (Fig. 4A, B). Age had been reported to be an important risk factor^33^, while male as a risk factor was mainly confirmed in AIDS-related KS in Africa^24^. Additionally, year of diagnosis later than 2008, White race, and localized lesions were associated with better OS (Fig. 3D, E, G). This may be due to patients diagnosed later 2008 receiving more systematic ART or more advanced treatment options^29^. Meanwhile, Royse et al. also reported that African Americans compared to white race were associated with lower 5-year overall survival (61.1% vs. 64.9%) and KS-specific survival (63.3% vs.75.5%)^34^. For patients with regional lesions, multiple lesions may mean worse OS (Fig. 4C). Spanish-Hispanic-Latino and marriage were not factors affecting OS (Fig. 3F, L). In terms of treatment, surgery and chemotherapy could improve the OS of patients with localized lesions and patients with metastatic lesions, respectively (Fig. 4D, F). It was considered that surgical excision should not be applied to extensive lesions, and repeated surgical excisions may cause severe functional impairment^24^. Systemic therapy was only to achieve disease control and symptom relief while maintaining quality of life. Accordingly, the recommended first-line agents were pegylated liposomal doxorubicin (PLD) and paclitaxel (PCT)^24^. The latest research also confirmed that paclitaxel plus ART could be used for the treatment of advanced AIDS-related KS with limited resources^13^. However, this analysis shown that whether radiotherapy did not affect the patient's OS (Fig. 4E). Other local therapies also included cryosurgery and laser^35^, isolated limb perfusion^4^ and local or intralesional chemical or immune-modifying agents^36^ and so on.

In this study, competition events accounted for about 26.3% of the total deaths, accounting for a very high proportion, which indicated that traditional Cox proportional hazards model may not be suitable for analyzing^20^. At the same time, total competition risk model analysis also proved that there was a potential competitive relationship between Non-KSSD and KSSD (Fig. 5A). Further analysis clarified that gender and Spanish-Hispanic-Latino did not affect the KSSD of patients (Fig. 5B, F). Meanwhile, the KSSD was higher for patients with the following characteristics: the diagnosis age was less than 60 years (Fig. 5C), the diagnosis year was earlier than 2008 (Fig. 5D), Black race (Fig. 5E), metastatic or regional lesions (Fig. 5G), with non-multifocal lesions (Fig. 5H), and single (Fig. 5L). For single patients (never married), it may mean a higher likelihood of homosexual sexual behavior, which may lead to the rapid progression of AIDS-related KS. And for most types of cancer, the survival rate decreases with age. But literature had confirmed that the prognosis of young KS patients with AIDS-related diseases was worse than that of elderly patients during the pre-HAART period^37^, which was consistent with our research results. As for treatment, surgery and radiotherapy improved the KS-specific survival of patients, but chemotherapy did not (Fig. 5I–K). And radiotherapy had been considered to be one of the most effective treatments for localized KS^38,39^.

Considering the difference in the prognosis among patients with cutaneous KS, we attempted to incorporate independent prognostic factors into the multiple competitive risk model to construct a nomogram that could predict the 5-, 10-, and 15-year KSSD (Fig. 6A). Meanwhile, compared with the model based on Cox proportional hazard regression (Fig. 6B), it had a higher C index (0.709 vs.0.625). As shown in Fig. 6, when predicting the prognosis of the same patient (Patient ID = 35767086), it showed a lower 5-year, 10-year, 15-year KSSD than the nomogram based on the Cox proportional hazard regression. This was because the model based on the Cox proportional hazard regression did not eliminate the bias caused by the competing events, thus overestimating the cumulative incidence^20,40^. Also, the evaluation of nomogram proved to have relatively high discrimination and calibration both in the training and validation cohorts (Fig. 7A–F), and showed good clinical practicability within an appropriate threshold probability range (Fig. 7G–I), which could assist doctors to make a personalized clinical decision for different cutaneous KS patients.

This study had some limitations that should not be ignored. Firstly, as a retrospective study, there were inherent selection biases and uncontrollable confounding factors. Secondly, this research was based on the SEER database of the United States and lacked external verification, so the results may not be completely applicable to other countries and populations. Thirdly, the SEER database lacked a description of the schemes of radiotherapy and chemotherapy, and the classification of KS subtypes, limited effective variables and rough analysis may limit the effects of this study. Fourthly, such as ART, low CD4 count had been shown to significantly affect the prognosis of KS^41,42^. However, the contribution of these variables was not evaluated in our study. This omission may have influenced the results, thereby limiting the best management strategy. Therefore, further studies were needed to clarify the effects of these factors on the prognosis and provide guidance for the treatment of cutaneous KS.

## Conclusion

This study was the first to analyze the prognosis of cutaneous KS based on a competing-risks model. This model revealed that radiotherapy and surgery could lower the KS-specific mortality, while chemotherapy and surgery could increase the OS of patients with metastatic and localized lesions, respectively. This validated nomogram provided individualized assessment and reliable prognostic prediction for cutaneous KS patients.

### Supplementary Information

Supplementary Figure 1.

Supplementary Legends.

## Data Availability

The datasets generated during and/or analyzed during the current study were available in the SEER repository, https://seer.cancer.gov/data.

## References

[1] Cesarman E (2019). Kaposi sarcoma. Nat. Rev. Dis. Primers.

[2] Vangipuram R, Tyring SK (2019). Epidemiology of Kaposi sarcoma: Review and description of the nonepidemic variant. Int. J. Dermatol..

[3] Etemad SA, Dewan AK (2019). Kaposi sarcoma updates. Dermatol. Clin..

[4] Boere T (2020). Isolated limb perfusion is an effective treatment modality for locally advanced Kaposi sarcoma of the extremities. Eur. J. Surg. Oncol..

[5] Rigo R, Lee A, Minkis K (2021). Nodular endemic Kaposi sarcoma successfully treated with Mohs micrographic surgery. Dermatol. Surg..

[6] Kaposi sarcoma. *Nat. Rev. Dis. Primers* **5**, 10 (2019). 10.1038/s41572-019-0065-4 30705285

[7] Shiels MS (2011). Cancer burden in the HIV-infected population in the United States. J. Natl. Cancer Inst..

[8] Ruocco E (2013). Kaposi's sarcoma: Etiology and pathogenesis, inducing factors, causal associations, and treatments: facts and controversies. Clin. Dermatol..

[9] Hong YK (2004). Lymphatic reprogramming of blood vascular endothelium by Kaposi sarcoma-associated herpesvirus. Nat. Genet.

[10] Yarchoan R, Uldrick TS (2018). HIV-Associated cancers and related diseases. N. Engl. J. Med..

[11] Ramaswami R (2019). A pilot study of liposomal doxorubicin combined with bevacizumab followed by bevacizumab monotherapy in patients with advanced Kaposi sarcoma. Clin. Cancer Res..

[12] Di Lorenzo G (2008). Activity and safety of pegylated liposomal doxorubicin as first-line therapy in the treatment of non-visceral classic Kaposi's sarcoma: a multicenter study. J. Investig. Dermatol..

[13] Krown SE (2020). Treatment of advanced AIDS-associated Kaposi sarcoma in resource-limited settings: a three-arm, open-label, randomised, non-inferiority trial. Lancet.

[14] Paydas S, Bagir EK, Deveci MA, Gonlusen G (2016). Clinical and prognostic significance of PD-1 and PD-L1 expression in sarcomas. Med. Oncol..

[15] Galanina N, Goodman AM, Cohen PR, Frampton GM, Kurzrock R (2018). Successful treatment of HIV-associated Kaposi sarcoma with immune checkpoint blockade. Cancer Immunol. Res..

[16] Maurichi A (2020). Factors affecting sentinel node metastasis in thin (T1) cutaneous melanomas: Development and external validation of a predictive nomogram. J. Clin. Oncol..

[17] Carmona-Bayonas A (2019). Prediction of progression-free survival in patients with advanced, well-differentiated, neuroendocrine tumors being treated with a somatostatin analog: The GETNE-TRASGU study. J. Clin. Oncol..

[18] Gray RJ (1988). A class of K-sample tests for comparing the cumulative incidence of a competing risk. Ann. Stat..

[19] Fine JP, Gray RJ (1999). A proportional hazards model for the subdistribution of a competing risk. J. Am. Stat. Assoc..

[20] Wolbers M, Koller MT, Witteman JC, Steyerberg EW (2009). Prognostic models with competing risks: Methods and application to coronary risk prediction. Epidemiology.

[21] Zuo Z (2021). Survival nomogram for stage IB non-small-cell lung cancer patients, based on the SEER database and an external validation cohort. Ann. Surg. Oncol..

[22] Kerr KF, Brown MD, Zhu K, Janes H (2016). Assessing the clinical impact of risk prediction models with decision curves: Guidance for correct interpretation and appropriate use. J. Clin. Oncol..

[23] Wu J (2020). A nomogram for predicting overall survival in patients with low-grade endometrial stromal sarcoma: A population-based analysis. Cancer Commun. (Lond.).

[24] Lebbe C (2019). Diagnosis and treatment of Kaposi's sarcoma: European consensus-based interdisciplinary guideline (EDF/EADO/EORTC). Eur. J. Cancer.

[25] Luo Q, Satcher Johnson A, Hall HI, Cahoon EK, Shiels M (2021). Kaposi sarcoma rates among persons living with human immunodeficiency virus in the United States: 2008–2016. Clin. Infect. Dis..

[26] Antman K, Chang Y (2000). Kaposi's sarcoma. N. Engl. J. Med..

[27] Polesel J (2010). Cancer incidence in people with AIDS in Italy. Int. J. Cancer.

[28] Franceschi S (2010). Changing patterns of cancer incidence in the early- and late-HAART periods: The Swiss HIV Cohort Study. Br. J. Cancer.

[29] Majaya E, Girdler-Brown BV, Muchengeti M, Singh E (2021). The impact of the South African antiretroviral treatment programme on the age-standardised incidence rate of Kaposi sarcoma, 1999–2016: An interrupted time series analysis. Int. J. Infect. Dis..

[30] Rubinstein PG, Aboulafia DM, Zloza A (2014). Malignancies in HIV/AIDS: From epidemiology to therapeutic challenges. Aids.

[31] Franceschi S, Arniani S, Balzi D, Geddes M (1996). Survival of classic Kaposi's sarcoma and risk of second cancer. Br. J. Cancer.

[32] van der Zwan JM (2012). Carcinoma of endocrine organs: results of the RARECARE project. Eur. J. Cancer.

[33] Guttman-Yassky E (2006). Classic Kaposi sarcoma. Which KSHV-seropositive individuals are at risk?. Cancer.

[34] Royse KE (2017). Disparities in Kaposi sarcoma incidence and survival in the United States: 2000–2013. PLoS ONE.

[35] Curatolo P, Careri R, Simioni A, Campana LG (2020). Cryotherapy, imiquimod, and electrochemotherapy are effective options for Kaposi sarcoma: A call for standardization to allow for comparisons and informed decisions. J. Cutan. Med. Surg..

[36] Brambilla L (2010). Intralesional vincristine as first-line therapy for nodular lesions in classic Kaposi sarcoma: A prospective study in 151 patients. Br. J. Dermatol..

[37] Stiller CA (2014). Descriptive epidemiology of Kaposi sarcoma in Europe. Report from the RARECARE project. Cancer Epidemiol..

[38] Singh NB, Lakier RH, Donde B (2008). Hypofractionated radiation therapy in the treatment of epidemic Kaposi sarcoma: A prospective randomized trial. Radiother. Oncol..

[39] Caccialanza M, Marca S, Piccinno R, Eulisse G (2008). Radiotherapy of classic and human immunodeficiency virus-related Kaposi's sarcoma: Results in 1482 lesions. J. Eur. Acad. Dermatol. Venereol..

[40] Li C (2021). Developing and validating a novel nomogram used a competing-risks model for predicting the prognosis of primary fallopian tube carcinoma: A retrospective study based on the SEER database. Ann. Transl. Med..

[41] Bohlius J (2014). Kaposi's Sarcoma in HIV-infected patients in South Africa: Multicohort study in the antiretroviral therapy era. Int. J. Cancer.

[42] Rohner E (2014). Incidence rate of Kaposi sarcoma in HIV-infected patients on antiretroviral therapy in Southern Africa: A prospective multicohort study. J. Acquir. Immune Defic. Syndr..

