# Supplementary figures and images for: Prognostic analysis of cutaneous Kaposi sarcoma based on a competing risk model
Source: Sci Rep. 2023 Oct 16;13:17572. doi: 10.1038/s41598-023-44800-5 (PMC10579376; doi:10.1038/s41598-023-44800-5)

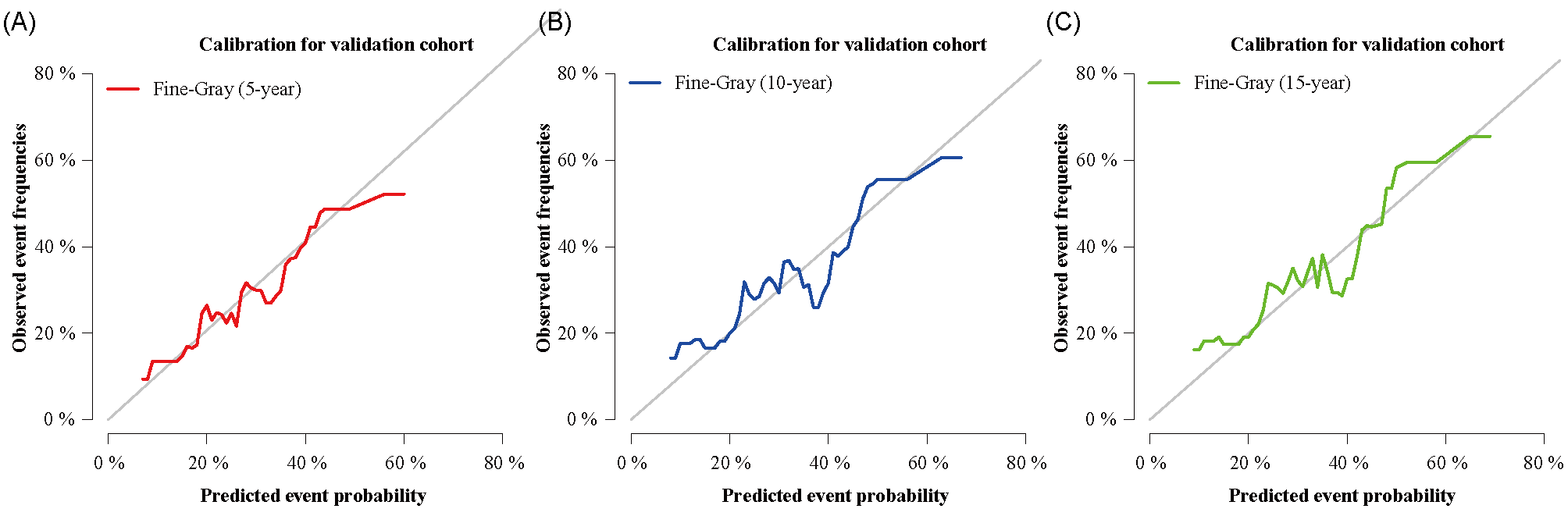

Supplement: Supplementary file 1 — Supplementary Figure 1. [file 41598_2023_44800_MOESM1_ESM.tif]
