# Supplementary material for: Prognostic analysis of cutaneous Kaposi sarcoma based on a competing risk model
Source: Sci Rep. 2023 Oct 16;13:17572. doi: 10.1038/s41598-023-44800-5 (PMC10579376; doi:10.1038/s41598-023-44800-5)
Supplement: Supplementary file 2 — Supplementary Legends. [file 41598_2023_44800_MOESM2_ESM.docx]

**Fig. S1.** A-C, the 5-, 10- and 15-year calibration curves for the validation cohort.
